# Long-term effect of a dietary intervention with two-healthy dietary approaches on food intake and nutrient density in coronary patients: results from the CORDIOPREV trial

**DOI:** 10.1007/s00394-022-02854-7

**Published:** 2022-03-29

**Authors:** Naomi Cano-Ibáñez, Gracia M. Quintana-Navarro, Juan F. Alcala-Diaz, Oriol A. Rangel-Zuñiga, Antonio Camargo, Elena M. Yubero-Serrano, Isabel Perez-Corral, Antonio P. Arenas-de Larriva, Antonio Garcia-Rios, Pablo Perez-Martinez, Javier Delgado-Lista, Jose Lopez-Miranda

**Affiliations:** 1 Department of Preventive Medicine and Public Health, University of Granada, 18071 Granada, Spain; 2 Consortium for Biomedical Research in Epidemiology and Public Health (CIBERESP), 28029 Madrid, Spain; 3 Instituto de Investigación Biosanitaria ibs.GRANADA, Complejo Hospitales Universitarios de Granada/Universidad de Granada, 18071 Granada, Spain; 4 Lipids and Atherosclerosis Unit, Internal Medicine Unit, Reina Sofia University Hospital, Av. Menendez Pidal s/n, 14004 Cordoba, Spain; 5 Maimonides Biomedical Research Institute of Cordoba (IMIBIC), 14014 Cordoba, Spain; 6 Department of Medical and Surgical Sciences, Faculty of Medicine and Nursing, University of Cordoba, 14014 Cordoba, Spain; 7 CIBER Fisiopatologia de la Obesidad y Nutricion (CIBEROBN), Instituto de Salud Carlos III, 28029 Madrid, Spain

**Keywords:** Mediterranean diet, Low-fat diet, Dietary intervention, Nutrient density, Dietary intake, Cardiovascular disease

## Abstract

**Background:**

Cardiovascular disease (CVD) is the leading cause of disease burden in the world by non-communicable diseases. Nutritional interventions promoting high-quality dietary patterns with low caloric intake value and high nutrient density (ND) could be linked to a better control of CVD risk and recurrence of coronary disease. This study aims to assess the effects of a dietary intervention based on MedDiet or Low-Fat dietary intervention over changes in ND and food intake after 1 and 7 years of follow-up of the CORDIOPREV study.

**Methods:**

We prospectively analyzed the results of the 802 coronary patients randomized to two healthy dietary patterns (MedDiet = 425, Low-Fat Diet = 377) who completed the 7 years of follow-up and had all the dietary data need. Dietary intake information obtained from a validated 137-item Food Frequency Questionnaire was used to calculate 1- and 7-year changes in dietary intake and ND (measured as nutrient intake per 1000 kcal). *T* test was used to ascertain differences in food intake and ND between groups across follow-up time. Within-subject (dietary allocation group) differences were analyzed with ANOVA repeated measures.

**Results:**

From baseline to 7 years of follow-up, significant increases of vegetables, fruits, and whole cereals within groups (*p* < 0.001) was found. We found a higher increase in dietary intake of certain food groups with MedDiet in comparison with Low-Fat Diet for vegetables (46.1 g/day vs. 18.1 g/day, *p* < 00.1), fruits (121.3 g/day vs. 72.9 g/day), legumes (4.3 g/day vs. 0.16 g/day) and nuts (7.3 g/day vs. − 3.7 g/day). There was a decrease in energy intake over time in both groups, slightly higher in Low-Fat Diet compared to MedDiet group (− 427.6 kcal/day vs. − 279.8 kcal/day at 1st year, and − 544.6 kcal/day vs. − 215.3 kcal/day after 7 years of follow-up). ND of all the nutrients increased within group across follow-up time, except for Saturated Fatty Acids (SFA), cholesterol and sodium (*p* < 0.001).

**Conclusions:**

A comprehensive dietary intervention improved quality of diet, reducing total energy intake and increasing the intake of healthy food groups and overall ND after 1 year and maintaining this trend after 7 years of follow-up. Our results reinforce the idea of the participation in trials, enhance nutrition literacy and produces better nutritional outcomes in adult patients with established CVD.

**Clinical trial registry:**

The trial was registered in 2009 at ClinicalTrials.gov (number NCT00924937).

## Introduction

Cardiovascular disease (CVD) is the leading cause of death in the world, accounting for a combined 18 million deaths in 2020 [1]. Despite of this rate, the number of patients with established CVD is growing in developed countries as a result of improved survival after acute vascular events and ageing population [2], which implies very high healthcare expenditures derived directly or indirectly from their care [3].

The risk of future cardiovascular events is higher in subjects with established CVD compared with those without this previous condition [4]. Recurrence of CVD is associated to unhealthy lifestyle habits, such as physical inactivity and poor dietary habits [5]. Regarding diet, potential favourable effects of healthy dietary patterns on CVD prevention have been ascribed to the intake of low energy-dense food groups as vegetables and fruits, legumes, whole-grains and nuts, instead of processed products and sugar-sweetened beverages providing the necessary amount of nutrients but with a low energy content [6, 7]. That is, not only the total energy is important, but also the nutritional value of the overall dietary pattern prescribed. The nutrient density (ND) is used as a measure of the quality of the diet, and it indicates the ratio of the nutrients present in a diet according to its caloric value [8].

Nutritional education interventions are comprehensive programmes based on healthy dietary behaviour deliver to patients aimed at improving patients’ clinical outcomes through the increase and maintenance of health behaviour [9]. Although some epidemiological studies as the INTERSTROKE case–control study [10] or Lyon study [11] showed that adherence to a healthy dietary pattern plays a preventive role in secondary CVD, to our knowledge, no large clinical trial on established CVD has assessed whether dietary intervention increases the quality of the food intake and ND after a long period of intervention.

The CORonary Diet Intervention with Olive oil and cardiovascular PREVention study (the CORDIOPREV study) is a large trial which randomized established CVD patients to follow a MedDiet (rich in olive oil) or a Low-Fat Diet for secondary prevention. The aim of the CORDIOPREV study is to compare the appearance of a composite of cardiovascular major events recurrence after an average follow-up of 7 years, supporting a causal association between adherence to both dietary patterns and diet quality over recurrence of coronary events [12].

The purpose of the present study was to assess the effects of a dietary intervention based on MedDiet or Low-Fat dietary intervention over changes in nutrient density (ND) and food intake after 1 and 7 years of follow-up of the CORDIOPREV study.

## Methods

### Design of the study

A detailed description of the design and methods of the CORonary Diet Intervention with Olive Oil and Cardiovascular PREVention (CORDIOPREV) study can be found elsewhere [12]. Briefly, the CORDIOPREV study was a 7-year unicentric, randomized, parallel-group, secondary prevention trial conducted in Spain, to compare 2 controlled dietary interventions: (a) Mediterranean-type diet, vs. (b) low-fat diet, on the risk of suffering new CVD.

### Ethics approval

The protocol was written in accordance with the principles of the Declaration of Helsinki. The respective Institutional Review Board (IRB) by the Human Investigation Review Committee of the Reina Sofia University Hospital (Córdoba, Spain) approved the study protocol. The trial was registered in 2009 at ClinicalTrials.gov (number NCT00924937). Recruitment took place from July 2009 to February 2012. All subjects provided written informed consent.

### Participants and data collection procedures.

**Fig. 1:**
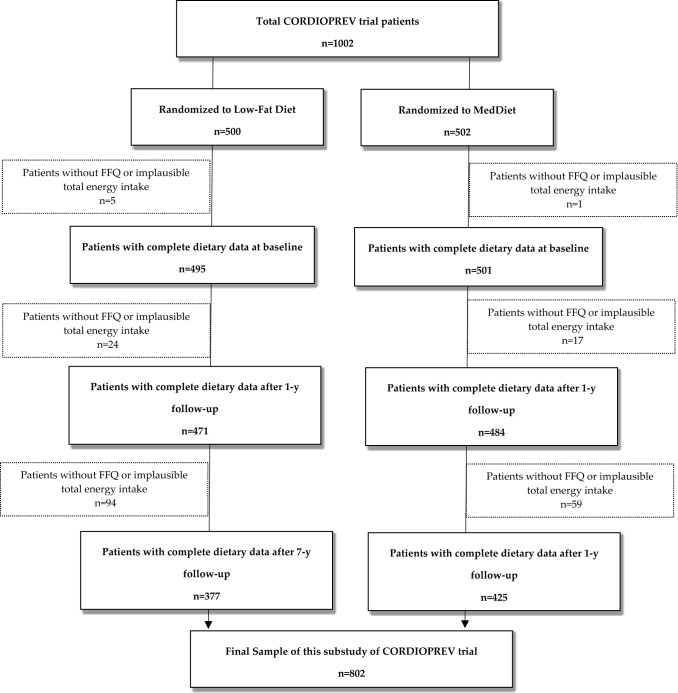
Flow chart of Participants from the CORDIOPREV trial included in this substudy

Eligible participants were men and women (aged over 20, but under 76 years) who had established CVD and without any clinical events in the previous 6 months, no severe illnesses or an expected life expectancy lower than the length of the study. From the 1002 participants recruited to the CORDIOPREV Study, we selected for the present longitudinal analysis those participants who completed at baseline and at 7 years of follow-up a Food Frequency Questionnaire (FFQ) and a Low-fat and Mediterranean Diet baseline adherence questionnaire. Those who failed to complete the FFQ were excluded from this sub-study. Among the available participants, we also excluded those individuals with extreme values for total energy intake in FFQ (< 800 kcal/day or > 4000 kcal/day for men; < 500 kcal/day or > 3500 kcal/day for women) according to the established criteria proposed by Willet et al. [13]. Finally, data from 802 participants were included in our analyses (Fig. 1). According to Fig. 1, 200 participants (around 20% of overall sample), did not complete the assessment of dietary intake after 7 years of follow-up. Considering that this attrition could introduce bias in our study, we performed an ancillary analyses comparing the characteristics of the participants without data at 7 years with those who completed the follow-up period.

### Dietary Intervention and adherence appraisal

Participants randomized to the MedDiet group were instructed to follow a Mediterranean Diet, with a minimum of 35% of calories as fat (22% Monounsaturated fat (MUFA), 6% Polyunsaturated fat (PUFA) and < 10% Saturated fat (SFA)), 15% proteins and a maximum of 50% carbohydrates. On the other hand, participants allocated in Low-Fat Diet followed a diet with less than 30% of total fat (< 10% SFA, 12–14% MUFA AND 6–8% PUFA), 15% protein and a minimum 55% carbohydrates. Trained dietitians conducted group sessions and individual motivational interview at inclusion and every 6 months. These sessions consisted of dietary counselling and informative talks reinforcing the dietary habits. No energy restriction and physical activity was promoted in any group. Prior the randomization and also yearly, adherence to each diet was appraised. The MedDiet was evaluated by a 14 item score previously validated tool [14] named MEDAS (MedDiet Adherence Score). Compliance with each of the 14 items relating to characteristic food habits was scored with 1 point if the goal was met or 0 points otherwise. Therefore, the total MEDAS range was 0–14, with 0 meaning no adherence and 14 meaning maximum adherence to Mediterranean Diet [15]. This tool was administered in both groups. To assess the adherence into low-fat diet, a 9-item dietary score was only administered to the participants allocated in this group. This screener was used in the PREDIMED (PREvención con DIeta MEDiterránea) study [16]. The total score ranged from 0 to 9.

More details on dietary intervention during the follow-up time are described and have been published in detail previously [12].

### Dietary intake assessment

Trained dieticians collected data on dietary intake at baseline and yearly basis during follow-up in a face-to-face interview. Dietary intake was assessed using a 137-item semi-quantitative FFQ previously and repeatedly validated in Spain [17]. The FFQ provides a list of foods and beverages commonly used by the Spanish population and asks about the consumption of these foods during the previous year. It includes 9 response options (never or almost never, 1–3 times a month, once a week, 2–4 times a week, 5–6 times a week, once a day, 2–3 times a week day, 4–6 times a day and more than 6 times a day). The indicated frequencies of consumption were converted to intakes per day and multiplied by the weight of the standard serving size to estimate the intake in grams per day. Nutrient information was derived from Spanish food composition tables [18, 19]. Thus, the dietary intake of a selection of nutrients including carbohydrates (CHO), total fat, monounsaturated (MUFAs), polyunsaturated (PUFAs) and saturated fatty acids (SFAs), cholesterol, protein, dietary fiber, vitamin A, all the B-vitamins, C, D, E and K, calcium, iron, phosphorus, magnesium, potassium, selenium, iodine, and zinc was assessed. The food intake of a selection of food groups including vegetables, fruits, dairy products, red/white meat, fish/seafood, nuts, legumes, bakery/sweets, olive oil and cereals/whole cereals was also assessed adjusting food groups intake by total energy intake [20].

To evaluate the ND of the diet, density intake of all nutrients aforementioned was calculated by dividing absolute nutrient intake by total energy intake. The nutrient density was expressed as nutrient intake per 1000 kcal [8]. Mean change was estimated for each nutrient as follows: (baseline nutrient density at 1 year—nutrient density at baseline) and similarly for 7-year follow-up. The procedure was equal for food groups. For the present analysis, we have included the changes in ND and food intake after 1 and 7 years of follow-up as dependent variables to ascertain the effect of dietary intervention on these changes.

### Covariate assessment

At baseline and once yearly, trained CORDIOPREV study staff collected information on lifestyle variables and sociodemographic data. The variables included were sex, age, smoking habit (non-smoker, current smoker or never smoker), and physical activity [21, 22]. Anthropometric variables (weight, height and waist circumference) were determined in accordance with the CORDIOPREV trial operations protocol. Weight and height were measured with calibrated scales and a wall-mounted stadiometer, respectively. Body Mass Index (BMI) was calculated as the weight in kilograms divided by the height in meters squared. Waist circumference (WC) was measured midway between the lowest rib and the iliac crest using an anthropometric tape.

### Statistical analysis

Qualitative variables were described as frequencies and percentages, *n* (%), whereas the quantitative variables were expressed as means and standard deviations (SD). *T* test was used to ascertain differences in food intake and ND between groups. To compare changes in dietary habits, analysis of variance (ANOVA) repeated measures within-subject variable (dietary group) was performed for each of the outcome measures. The significance level was set at 5%. All analyses were performed using Stata software (15.0, StataCorp LP, Tx. USA).

## Results

### Characteristics of study patients at baseline according to dietary intervention group

The baseline characteristics of participants according to dietary intervention group are presented in Table 1. The current study included a total of 802 participants (673 men) with an average age of 59.2 years at baseline. Both groups showed similar socio-demographic and clinical characteristics except for diabetes condition (being slightly higher in low-fat diet group). The ancillary analysis comparing the baseline characteristics of the participants without data at 7 years with those who completed the follow-up assessment is shown in Table 4 Appendix. There were no differences between groups for any sociodemographic or lifestyle variables, (*p* > 0.05) except for educational level.

**Table 1:** Baseline characteristics of the CORDIOPREV study participants according to dietary intervention of the population with all data available included in this article (*n* = 802)

	Mediterranean diet		Low-fat diet		*p *value
*n*	425		377		
	*n*	(%)	*n*	(%)	
Sex					0.945
Men	357	(84.0)	316	(83.8)	
Women	68	(16.0)	61	(16.2)	
Family history of premature CHD					0.472
No	364	(85.7)	316	(83.8)	
Yes	61	(14.4)	61	(16.2)	
Smoking habit					0.578
Never smoker	124	(29.2)	100	(26.5)	
Former smoker	265	(62.4)	239	(63.4)	
Current smoker	36	(8.5)	38	(10.1)	
Hypertension					0.955
No	139	(32.7)	124	(32.9)	
Yes	286	(67.3)	253	(67.1)	
Dyslipidaemia					0.463
LdL < 100 mg/dL	301	(70.8)	258	(68.4)	
LdL > = 100 mg/dL	124	(29.2)	119	(31.6)	
Diabetes					
No	217	(51.1)	163	(43.2)	**0.027**
Yes	208	(48.9)	214	(56.8)	
Marital status					0.563
Married	363	(85.4)	329	(87.3)	
Others^a^	53	(12.5)	46	(12.2)	
Missing value	9	(2.1)	2	(0.5)	
Educational level					0.246
Primary education	281	(66.1)	263	(69.8)	
Secondary education	86	(20.2)	78	(20.7)	
University education	43	(10.1)	26	(6.9)	
Missing Value	15	(3.6)	10	(2.6)	

	Mediterranean diet		Low-fat diet		*p* value
***n***	425		377		
	Mean	(SD)	Mean	(SD)	
Age (years)	59.2	(9.2)	59.2	(8.2)	0.929
BMI (kg/m^2^)	31.0	(4.4)	31.2	(4.5)	0.557
Adherence to MedDiet (points)	8.9	(2.0)	8.7	(1.8)	0.923
Adherence to low-fat diet (points)	3.9	(1.5)	3.9	(1.6)	0.912
Physical activity (METs-min/day)	193.3	(235.7)	186.1	(212.0)	0.699

Values are presented as means ± SD for continuous variables and *n* (%) for categorical variables. Pearson’s *χ*^2^ test was performed for categorical variables and *T* student test for continuous variables.

*BMI*: Body Mass Index

Others^a^: Includes single, widowed and divorced.

### Changes in dietary sources according to dietary intervention group after 1 and 7 years of follow-up

Table 2 shows changes in food sources according to randomization group. The dietary intake of vegetables, fruits, and whole cereals significantly increased within groups across follow-up period (< 0.001). Meanwhile, the mean intake of dairy products, bakery/sweets, cereals and red meat decreased in both groups compared to baseline intake (< 0.001). Differences between groups were notorious after 7-year follow-up. Some of the greatest increases in dietary intake were observed in MedDiet in comparison with Low-Fat Diet for vegetables (46.1 g/day vs. 18.1 g/day, *p* < 00.1), fruits (121.3 g/day vs. 72.9 g/day), legumes (4.3 g/ day vs. 0.16 g/day) and nuts (7.3 g/day vs. − 3.7 g/day). The relatives mean % changes after follow-up are represented in Fig. 1. The largest changes (more than 20%) were observed for the intakes of fruits and legumes, being the increased intake upper at 7 years of follow-up compared to 1st year in both groups.

**Table 2:** Mean values and mean changes in dietary sources according to dietary pattern at baseline and after 1 and 7 years of follow-up in the CORDIOPREV study (*n* = 802)

	MedDiet	Low-fat diet	*p* value between group
Vegetable, Mean (SD), (g/day)			
Baseline	261.8 (94.5)	254.3 (99.4)	0.276
1-year change, (95%, CI)	− 16.0 (− 27.1, − 5.0)	0.9 (− 11.4, 13.3)	**0.044**
7-year change, (95%, CI)	46.1 (35.4, 56.7)	18.1 (6.0, 30.2)	**0.001**
*p* value within group	** < 0.001**	** < 0.001**	
Fruits, Mean (SD), (g/day)			
Baseline	346.3 (181.3)	343.2 (191.2)	0.815
1-year change, (95%, CI)	39.3 (17.4, 61.3)	27.7 (6.8, 48.7)	0.456
7-year change, (95%, CI)	121.3 (99.7, 142.8)	72.9 (50.3, 95.5)	**0.002**
* p* value within group	** < 0.001**	** < 0.001**	
Legumes, Mean (SD), (g/day)			
Baseline	22.6 (11.8)	23.9 (17.3)	0.206
1-year change, (95%, CI)	3.4 (1.7, 5.1)	− 1.5 (− 3.7, 0.7)	**0.001**
7-year change, (95%, CI)	4.3 (2.9, 5.8)	0.16 (− 1.9, 2.2)	**0.001**
* p* value within group	** < 0.001**	** < 0.001**	
Dairy, Mean (SD), (g/day)			
Baseline	387.0 (191.5)	351.3 (186.8)	**0.008**
1-year change, (95%, CI)	− 27.9 (− 47.2, − 8.6)	15.2 − 3.6, 34.1)	**0.002**
7-year change, (95%, CI)	− 68.6 (− 88.8, − 48.3)	− 24.3 (− 46.1, − 2.5)	**0.004**
* p* value within group	** < 0.001**	** < 0.001**	
Bakery and Sweets, Mean (SD), (g/day)			
Baseline	26.9 (24.7)	27.4 (26.0)	0.793
1-year change, (95%, CI)	− 9.8 (− 12.2, − 7.4)	0.2 (− 3.0, 3.3)	** < 0.001**
7-year change, (95%, CI)	− 15.8 (− 18.1, − 13.6)	− 13.2 (− 15.9, − 10.4)	0.134
* p* value within group	** < 0.001**	** < 0.001**	
Cereals, Mean (SD), (g/day)			
Baseline	176.8 (80.5)	182.8 (86.1)	0.310
1-year change, (95%, CI)	− 23.3 (− 32.2, − 14.4)	− 35.1 (− 45.2, − 25.0)	0.085
7-year change, (95%, CI)	− 36.9 (− 45.7, − 28.0)	− 41.3 (− 51.7, − 31.0)	0.515
* p* value within group	** < 0.001**	** < 0.001**	
Whole Cereals, Mean (SD), (g/day)			
Baseline	40.1 (72.3)	43.9	0.481
1-year change, (95%, CI)	23.2 (14.5, 31.8)	19.4 (9.9, 29.0)	0.567
7-year change, (95%, CI)	6.9 (− 1.5, 15.4)	2.6 (− 7.3, 12.4)	0.507
* p* value within group	** < 0.001**	** < 0.001**	
Fish and seafood, Mean (SD), (g/day)			
Baseline	106.0 (47.2)	102.9 (46.3)	0.361
1-year change, (95%, CI)	− 19.3 (− 24.5, − 14.1)	− 26.3 (− 31.2, − 21.4)	0.056
7-year change, (95%, CI)	− 7.8 (− 12.6, − 3.0)	− 22.6 (− 27.7, − 17.6)	** < 0.001**
* p* value within group	** < 0.001**	** < 0.001**	
Red Meat, Mean (SD), (g/day)			
Baseline	40.7 (31.1)	46.8 (35.5)	0.102
1-year change, (95%, CI)	− 23.0 (− 25.9, − 20.1)	− 23.8 (− 27.3, − 20.3)	0.737
7-year change, (95%, CI)	− 24.0 (− 27.1, − 21.0)	− 25.9 (− 29.5, − 22.3)	0.439
* p* value within group	** < 0.001**	** < 0.001**	
White Meat, Mean (SD), (g/day)			
Baseline	69.7 (34.4)	72.3 (37.5)	0.304
1-year change, (95%, CI)	19.6 (10.0, 29.3)	5.0 (− 3.1, 13.1)	**0.025**
7-year change, (95%, CI)	− 60.2 (− 65.0, − 55.4)	− 60.3 (− 65.7, − 54.9)	0.980
* p* value within group	** < 0.001**	** < 0.001**	
Nuts, Mean (SD), (g/day)			
Baseline	9.3 (10.7)	8.5 (10.5)	0.289
1-year change, (95%, CI)	2.1 (0.8, 3.3)	− 3.8 (− 4.8, − 2.7)	** < 0.001**
7-year change, (95%, CI)	7.3 (5.9, 8.7)	− 3.7 (− 4.9, − 2.5)	** < 0.001**
* p* value within group	** < 0.001**	** < 0.001**	
Olive oil, Mean (SD), (g/day)			
Baseline	34.9 (12.8)	33.4 (12.3)	0.099
1-year change, (95%, CI)	5.2 (3.6, 6.8)	− 14.0 (− 15.5, − 12.4)	** < 0.001**
7-year change, (95%, CI)	12.9 (11.5, 14.4)	− 11.7 (− 13.3, − 10.1)	** < 0.001**
* p* value within group	** < 0.001**	** < 0.001**	

Values are presented as means and standard deviations (SD) at baseline point and changes (95% CI) at 1 and 7 years of follow-up. *p* value for differences between groups at 1- and 7-year follow-up using *t* test. *p* value for differences within group using ANOVA for repeated measure. Values in bold showed significant association

*CI* Confidence Intervals, *MedDiet* Mediterranean Diet

### Changes in ND according to dietary intervention group after 1 and 7 years of follow-up

**Fig. 2:**
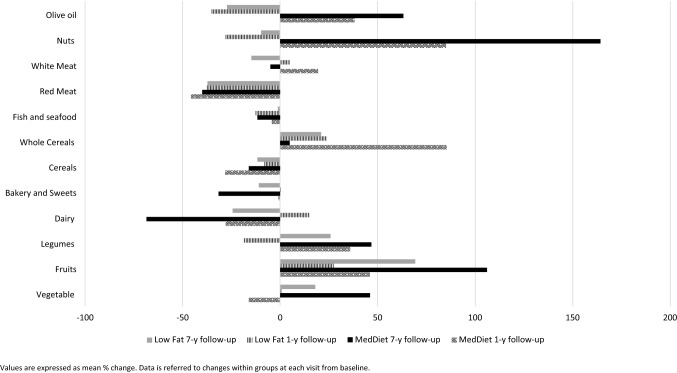
Relatives changes in food intake after 1- and 7-year follow-up according to randomization group in the CORDIOPREV study

**Fig. 3:**
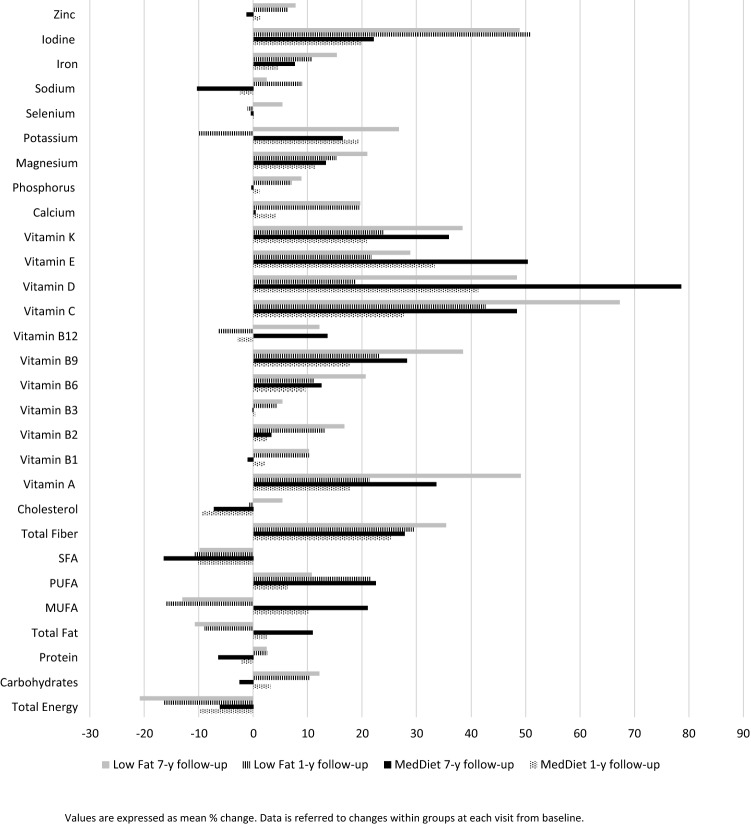
Relatives changes in total energy and nutrient density after 1- and 7-year follow-up according to randomization group in the CORDIOPREV study

There was a decrease in energy intake over time in both groups, slightly higher in Low-Fat Diet compared to MedDiet group (− 427.6 kcal/day vs. − 279.8 kcal/day at 1st year, and − 544.6 kcal/day vs. − 215.3 kcal/day after 7 years of follow-up) (Table 3). ND of all the nutrients increased within group across follow-up time, except for SFA, cholesterol, vitamin B12 and sodium (*p* < 0.001). Taking into account the differences between participants, those allocated in MedDiet compared with the respective counterparts, exhibited higher ND of total fat and quality subtypes of dietary fat (MUFA and PUFA), vitamin D and E through the follow-up study (*p* < 0.001). By contrary, Low-Fat Diet participants displayed higher ND for vitamin B9, calcium, phosphorus, magnesium, potassium, selenium, iodine, iron and zinc (*p* < 0.05). The relatives mean % changes of food intake, ND and total energy are represented in Figs. 2 and 3, respectively**.** ND increases after 1 year as compared to baseline, although the increase was larger after 7 years. The largest changes were found for iodine, potassium, vitamin K, E, D, C, A, total fiber and PUFA. According to food groups, red meat intake decreases in both groups; meanwhile, the intake of fruits, vegetables and legumes increases in both groups after the dietary intervention, maintaining this trend over time. Significantly, the largest changes were found for nuts and olive oil in the MedDiet group.

**Table 3:** Mean values and changes in nutrient density according to dietary pattern at baseline and after 1 and 7 years of follow-up in the CORDIOPREV study (*n* = 802)

	MedDiet	Low-fat diet	*p* value between group
Energy, Mean (SD), (kcal/day)			
Baseline	2247.8 (498.7)	2266.1 (523.9)	0.613
1-year change, (95%, CI)	− 279.8 (− 327.5, − 232.0)	− 427.6 (− 479.2, − 375,9)	** < 0.001**
7-year change, (95%, CI)	− 215.3 (− 262.2, − 168.3)	− 544.6 (− 599.4, − 489.9)	** < 0.001**
* p* value within group	** < 0.001**	** < 0.001**	
Carbohydrates, Mean (SD), (g/1000 kcal)			
Baseline	103.5 (16.1)	103.8 (16.7)	0.802
1-year change, (95%, CI)	1.0 (− 0.9, 2.9)	8.6 (6.5, 10.7)	** < 0.001**
7-year change, (95%, CI)	− 4.9 (− 6.8, − 3.0)	9.9 (7.8, 11.9)	** < 0.001**
* p* value within group	** < 0.001**	** < 0.001**	
Protein, Mean (SD), (g/1000 kcal)			
Baseline	46.3 (6.7)	46.4 (7.3)	0.814
1-year change, (95%, CI)	− 1.7 (− 2.4, − 0.9)	0.6 (− 0.2, 1.4)	** < 0.001**
7-year change, (95%, CI)	− 3.7 (− 4.4, − 3.0)	0.3 (− 0.5, 1.2)	** < 0.001**
* p* value within group	** < 0.001**	** < 0.001**	
Total Fat, Mean (SD), (g/1000 kcal)			
Baseline	41.4	40.9	0.241
1-year change, (95%, CI)	0.2 (− 0.6, 1.0)	− 4.3 (− 5.2, − 3.5)	** < 0.001**
7-year change, (95%, CI)	3.6 (2.7, 4.4)	− 5.2 (− 6.0, − 4.3)	** < 0.001**
* p* value within group	** < 0.001**	** < 0.001**	
MUFA, Mean (SD), (g/1000 kcal)			
Baseline	20.4 (3.9)	19.9 (3.7)	0.080
1-year change, (95%, CI)	1.4 (0.9, 1.9)	− 3.6 (− 4.1, − 3.1)	** < 0.001**
7-year change, (95%, CI)	3.5 (2.9, 4.0)	− 3.1 (− 3.6, − 2.6)	** < 0.001**
* p* value within group	** < 0.001**	** < 0.001**	
PUFA, Mean (SD), (g/1000 kcal)			
Baseline	7.1 (1.9)	7.0 (1.9)	0.327
1-year change, (95%, CI)	0.1 (− 0.1, 0.4)	1.1 (0.8, 1.5)	** < 0.001**
7-year change, (95%, CI)	1.1 (0.9, 1.4)	0.3 (0.1, 0.6)	** < 0.001**
*p* value within group	** < 0.001**	** < 0.001**	
SFA, Mean (SD), (g/1000 kcal)			
Baseline	9.9 (2.0)	9.9 (2.1)	0.961
1-year change, (95%, CI)	− 1.2 (− 1.4, − 1.0)	− 1.3 (− 1.6,− 1.1)	0.586
7-year change, (95%, CI)	− 1.8 (− 2.1, − 1.6)	− 1.3 (− 1.6, − 0.9)	0.009
*p* value within group	** < 0.001**	** < 0.001**	
Total Fiber, Mean (SD), (g/1000 kcal)			
Baseline	11.3 (3.3)	11.4 (3.4)	0.751
1-year change, (95%, CI)	2.1 (1.7, 2.4)	2.6 (2.1, 3.0)	0.079
7-year change, (95%, CI)	2.2 (1.9, 2.6)	3.1 (2.6, 3.5)	**0.005**
*p* value within group	** < 0.001**	** < 0.001**	
Cholesterol, Mean (SD), (g/1000 kcal)			
Baseline	147.9 (35.9)	147.9 (36.4)	0.981
1-year change, (95%, CI)	− 18.5 (− 22.4, − 14.6)	− 7.1 (− 11.8, − 2.4)	**0.001**
7-year change, (95%, CI)	− 17.0 (− 20.8, − 13.3)	0.2 (− 4.2, 4.6)	** < 0.001**
* p* value within group	** < 0.001**	** < 0.001**	
Vitamin A, Mean (SD), (µg/1000 kcal)			
Baseline	341.5 (196.7)	333.7 (214.0)	0.593
1-year change, (95%, CI)	− 18.7 (− 56.6, 19.2)	4.2 (− 21.1, 29.6)	0.335
7-year change, (95%, CI)	35.7 (9.8, 61.7)	61.8 (31.9, 91.8)	0.194
*p* value within group	** < 0.001**	** < 0.001**	
Vitamin B1, Mean (SD), (mg/1000 kcal)			
Baseline	0.7 (0.1)	0.7 (0.1)	0.794
1-year change, (95%, CI)	0.1 (− 0.01, 0.10)	0.1 (0.04, 0.70)	** < 0.001**
7-year change, (95%, CI)	− 0.1 (− 0.04, − 0.10)	0.1 (0.04, 0.70)	** < 0.001**
*p* value within group	** < 0.001**	** < 0.001**	
Vitamin B2, Mean (SD), (mg/1000 kcal)			
Baseline	0.9 (0.2)	0.9 (0.2)	0.321
1-year change, (95%, CI)	0 (− 0.03, 0.02)	0.1 (0.07, 0.11)	** < 0.001**
7-year change, (95%, CI)	0 (0.04, 0.07)	0.1 (0.01, 0.14)	** < 0.001**
*p* value within group	** < 0.001**	** < 0.001**	
Vitamin B3, Mean (SD), (mg/1000 kcal)			
Baseline	18.6 (3.4)	18.8 (3.7)	0.528
1-year change, (95%, CI)	− 0.3 (− 0.72, 0.03)	0.4 (− 0.03, 0.75)	0.010
7-year change, (95%, CI)	− 0.5 (− 0.89, − 0.15)	0.6 (0.18, 1.05)	** < 0.001**
*p* value within group	** < 0.001**	** < 0.001**	
Vitamin B6, Mean (SD), (mg/1000 kcal)			
Baseline	1.0 (0.2)	1.0 (0.2)	0.293
1-year change, (95%, CI)	0.1 (0.04, 0.09)	0.1 (0.06, 0.11)	0.299
7-year change, (95%, CI)	0.1 (0.06, 0.11)	0.2 (0.14, 0.20)	** < 0.001**
*p* value within group	** < 0.001**	** < 0.001**	
Vitamin B9, Mean (SD), (mg/1000 kcal)			
Baseline	144.5 (40.0)	142.6 (40.1)	0.507
1-year change, (95%, CI)	17.0 (12.61, 21.33)	25.3 (20.62, 30.00)	0.011
7-year change, (95%, CI)	29.3 (24.79, 33.74)	41.6 (36.23, 46.96)	**0.001**
* p* value within group	** < 0.001**	** < 0.001**	
Vitamin B12, Mean (SD), (mg/1000 kcal)			
Baseline	3.8 (1.2)	3.8 (1.3)	0.980
1-year change, (95%, CI)	− 0.1 (− 0.27, 0.06)	− 0.2 (− 0.39, − 0.09)	0.232
7-year change, (95%, CI)	0.2 (0.07, 0.35)	0.1 (− 0.08, 0.25)	0.279
* p* value within group	** < 0.001**	** < 0.001**	
Iron, Mean (SD), (mg/1000 kcal)			
Baseline	6.9 (1.0)	6.9 (1.0)	0.621
1-year change, (95%, CI)	0.2 (0.09, 0.32)	0.7 (0.55, 0.77)	** < 0.001**
7-year change, (95%, CI)	0.4 (0.27, 0.51)	0.9 (0.82, 1.07)	** < 0.001**
*p* value within group	** < 0.001**	** < 0.001**	
Vitamin C, Mean (SD), (mg/1000 kcal)			
Baseline	83.6 (34.8)	82.6 (34.1)	0.672
1-year change, (95%, CI)	8.8 (4.6, 13.0)	17.3 (12.6, 21.9)	0.008
7-year change, (95%, CI)	22.7 (18.9, 26.5)	31.1 (26.6, 35.6)	**0.005**
*p* value within group	** < 0.001**	** < 0.001**	
Vitamin D, Mean (SD), (µg/1000 kcal)			
Baseline	3.5 (1.8)	3.4 (2.0)	0.380
1-year change, (95%, CI)	0.1 (− 0.1, 0.3)	− 0.7 (− 0.9, − 0.5)	** < 0.001**
7-year change, (95%, CI)	0.9 (0.6, 1.1)	− 0.1 (− 0.4, 0.1)	** < 0.001**
*p* value within group	** < 0.001**	** < 0.001**	
Vitamin E, Mean (SD), (mg/1000 kcal)			
Baseline	8.4 (2.39	7.9 (2.3)	**0.004**
1-year change, (95%, CI)	1.8 (1.5, 2.1)	1.0 (0.6, 1.3)	**0.001**
7-year change, (95%, CI)	3.2 (2.9, 3.4)	1.3 (1.0, 1.6)	** < 0.001**
*p* value within group	** < 0.001**	** < 0.001**	
Vitamin K, Mean (SD), (µg/1000 kcal)			
Baseline	136.0 (60.7)	129.6 (63.4)	0.142
1-year change, (95%, CI)	5.3 (− 1.8, 12.3)	11.5 (3.7, 19.3)	0.243
7-year change, (95%, CI)	15.9 (8.6, 23.1)	19.3 (10.9, 27.6)	0.542
*p* value within group	** < 0.001**	** < 0.001**	
Calcium, Mean (SD), (mg/1000 kcal)			
Baseline	451.5 (126.4)	433.1 (125.7)	0.040
1-year change, (95%, CI)	0.7 (− 12.4, 13.8)	65.1 (50.0, 80.2)	** < 0.001**
7-year change, (95%, CI)	− 21.1 (− 34.6, − 7.7)	58.4 (42.3, 74.5)	** < 0.001**
*p* value within group	** < 0.001**	** < 0.001**	
Phosphorus, Mean (SD), (mg/1000 kcal)			
Baseline	746.9 (127.6)	745.7 (129.0)	0.890
1-year change, (95%, CI)	− 3.8 (− 17.5, 9.9)	42.7 (28.4, 56.9)	< 0.001
7-year change, (95%, CI)	− 18.1 (− 31.4, − 4.8)	50.7 (35.3, 66.0)	< 0.001
*p* value within group	** < 0.001**	** < 0.001**	
Magnesium, Mean (SD), (mg/1000 kcal)			
Baseline	165.3 (32.8)	165.1 (32.6)	0.909
1-year change, (95%, CI)	13.9 (10.2, 17.7)	21.0 (17.1, 24.9)	0.011
7-year change, (95%, CI)	16.1 (12.3, 20.0)	28.7 (24.4, 33.0)	** < 0.001**
*p* value within group	** < 0.001**	** < 0.001**	
Potassium, Mean (SD), (µg/1000 kcal)			
Baseline	1674.6 (333.8)	1653.7 (322.2)	0.369
1-year change, (95%, CI)	107.7	274.1	** < 0.001**
7-year change, (95%, CI)	217.2	380.2	** < 0.001**
*p* value within group	** < 0.001**	** < 0.001**	
Selenium, Mean (SD), (mg/1000 kcal)			
Baseline	56.8 (10.9)	57.0 (10.8)	0.841
1-year change, (95%, CI)	− 1.4 (− 2.7, − 0.1)	− 1.5 (− 2.8, − 0.2)	0.920
7-year change, (95%, CI)	− 2.0 (− 3.3, − 0.8)	1.3 (− 0.1, 2.7)	**0.001**
*p* value within group	** < 0.001**	** < 0.001**	
Sodium, Mean (SD), (µg/1000 kcal)			
Baseline	1191.7 (225.6)	1201.3 (235.5)	0.558
1-year change, (95%, CI)	− 54.0 (− 82.4, − 25.6)	72.3 (41.8, 102.7)	** < 0.001**
7-year change, (95%, CI)	− 154.8 (− 180.1, − 129.5)	− 9.7 (− 39.8, 20.4)	** < 0.001**
*p* value within group	** < 0.001**	** < 0.001**	
Iodine, Mean (SD), (mg/1000 kcal)			
Baseline	143.4 (75.0)	135.7 (72.5)	**0.140**
1-year change, (95%, CI)	1.7 (− 6.8, 10.3)	33.6 (24.9, 42.4)	** < 0.001**
7-year change, (95%, CI)	− 2.9 (− 11.1, 5.3)	29.4 19.4, 39.3)	** < 0.001**
*p* value within group	** < 0.001**	** < 0.001**	
Zinc, Mean (SD), (µg/1000 kcal)			
Baseline	5.7 (1.0)	5.8 (1.0)	0.242
1-year change, (95%, CI)	− 0.1 (− 0.2, 0.1)	0.2 (0.1, 0.4)	**0.001**
7-year change, (95%, CI)	− 0.2 (− 0.3, − 0.1)	0.3 (0.2, 0.4)	** < 0.001**
*p* value within group	** < 0.001**	** < 0.001**	

Values are presented as means and standard deviations (SD) at baseline point and changes (95% CI) at 1 and 7 years of follow-up. *p* value for differences between groups at 1- and 7-year follow-up using *t* test. *p* value for differences within group using ANOVA for repeated measure. Values in bold showed significant association

*CI* Confidence Intervals, *MedDiet* Mediterranean Diet

## Discussion

In the present study, we evaluated the association between dietary intervention promoting two healthy dietary patterns on changes in food and ND intake in a population of persons in cardiovascular secondary prevention. The main findings are twofold: first, dietary intervention improves quality of diet, increasing the intake of healthy foods and the ND and reducing the energy intake in both groups. Second, the aforementioned enhancement persists through long-term follow-up, with the improvements lasting for 7 years, independently of randomization group.

As far as we know, this is the first study to present data on intra and inter-individual changes in diet quality over a large sample of population with established CVD after a long follow-up period time. The CORDIOPREV participants stayed in contact with the research staff receiving individualized nutritional counselling and reinforcing food choices. These interventions have been related to dietary intake changes, with a markedly increased of healthy food as vegetables, fruits and whole cereals and a decrease of undesirable ones as bakery/sweets and red meat in both groups. These findings are consistent with the previously reported preliminary findings of this trial [23] and similar to a recent interim analysis of the ongoing PREDIMED-Plus trial [24].

Although some studies question the feasibility of having a large number of dietary changes during the follow-up dietary intervention [25], our findings support the idea that the overall mentioned changes in food intakes were at least maintained through follow-up. Probably, this significant maintenance of behavioural changes were only possible by the perseverance in the level of dietary intervention, and focusing on individual’s health knowledge and nutrition literacy [26]. Other factors may also contribute to explain the high retention rates and long-term dietary adherence in our study. One of them could be attributable to the health characteristic of participants included in the study. It is possible that CORDIOPREV trial participants were aware of their coronary problems and its relationship with poor food habits, and thus firmly motivated to comply with dietary advices. Contrary to our results, other studies have reported that improvements in diet quality appears in the short term, declining after a long follow-up period [27–29]. All of these studies are pointing out a trend toward worsening diet quality across follow-up. Nevertheless, these studies evaluated the changes after short follow-up time, usually 6, 12 or 24 months after intervention with different settings and populations. In fact, dietary interventions with a so long follow-up are really scarce, and one of the added values of this article is that it shows that, with an adequate intervention, chronic maintenance of dietary adherence and long-term change of dietary habits is possible (which had not been well established until now).

Even though the CORDIOPREV dietary intervention did not promoted energy restriction, the energy intake decreased significantly in both groups, being the energy reduction slight higher in Low-Fat Diet group, probably attributable to its low fat content [30]. In this line of thought, dietary quality has been broadly defined by the ratio energy/nutrient intake, being inversely linked between them [31]. Based on our findings, at the same time that the energy intake decreased, the ND of the majority of the vitamins and minerals increased in both groups with reference to baseline point. Between the different healthy dietary patterns assessed, the MedDiet group showed relatively better ND compared to the other group, specifically for MUFA, PUFA, Vitamins D and E. This result could be explained by the higher intake of some specified food groups as nuts and olive oil, foods richest in these nutrients, and the hallmark of MedDiet [32]. A recent study including high risk CVD adults [33] that evaluated the effect of improving MedDiet adherence on ND after 1 year of follow-up reported similar findings. However, their results should be carefully interpreted, because although they examined the same cohort over time, the follow-up time was shorter and they not compared to another dietary group as we do. Regarding secondary prevention, it is well documented that requires a multidisciplinary approach, highlighting the role of dietary intervention. Dietary patterns with low caloric value and high ND are key in the prevention of its recurrence [34]. These findings highlight that the changes in dietary quality are present and can be maintained after a long dietary intervention. The following step is to assess if these dietary changes could be part of several effect in health, as a decreased risk of CVD recurrence and development of other chronic diseases. Although the energy reduction and ND for some minerals is higher in Low-Fat Diet allocation group, is important to note that anyone enrolled in the CORDIOPREV study, have obtained a nutritional benefit. This idea reinforces not only the fact that nutritional counselling improves quality of diet, but also that the participation in this trial, independently of allocation group, enhanced nutrition literacy and produced better nutritional outcomes.

The current study has some limitations that need to be addressed. First, the study sample is not representative of the general population, since only adults with established CVD were included, being the results not extrapolated to the overall population. Nevertheless, our population represents an important proportion of current adult Western societies. Second, other possible determinants of ND might have not been evaluated in this study; however, the most prominent sociodemographic and lifestyle factors in the literature have been collected. Third, although we used a FFQ to measure dietary intakes validated in adult Spanish individuals with good reproducibility and validity [35], it might not be the ideal tool to measure micronutrient intake [36]. Fourth, the use of FFQ could include a memory bias and, we could not rule out the possibility that intake of some nutrients have been misclassified. However, because we are using the same tool and in the same way, the potential measurement errors should be constant in magnitude and direction, so we think that our estimates of differences are still valid. Furthermore, there may be concern that FFQ responses may change simply as a result of the advice given, rather than reflecting real change. One way to check this would have involved using some objective laboratory measures of nutritional components, something that we did not have in our study, for correlation with FFQ scores. However, the FFQ employed in our study is a validated tool with a high reproducibility [17]. Reproducibility reflects reliability and refers to the similarity of the same method at different timepoints [37], so FFQ is a tool that is established to be responsive to change and the observed differences are reliable. Fifth, there may be an impact of learning bias arising from repeated FFQ measurements. Participation in clinical trials may improve outcomes due to the simple fact of being monitored within the study [38]. The possibility of learning bias may arise due to repetition of FFQs during the follow-up period. This could result in improvements in the dietary changes reported by control participants due to their increased knowledge of healthy living. This bias may lead to a negative study, like ours. In our defence, FFQ validation would have ensured responsiveness to change [37]. Critics may argue that this validation may not map well onto out repetition and follow-up time frame. However, in other studies that have address the same question as ours [33, 39] with similar FFQ repetition and follow-up with emphasis on healthy living, have shown positive findings. This means that in these studies the control group responses were not influenced by repeated FFQ measurements. While learning bias is possible, it is unlikely that it materially impacted on control group responses compared to intervention.

Sixth, after 7 years of follow-up, we had a decrease of around 20% of our overall sample, a feature that may introduce bias depending on the numbers and the manner of the loss. Therefore, we performed an ancillary analysis (Table 4 Appendix), where we demonstrated that, except for one of 13 variables, there weren´t any differences between groups. Give this lack of systematic difference, it is reasonable to assume that results of the complete data set are trustworthy.

Seventh, increasing average age of the participants during the 7-year course of the trial may impact on changes in diet independent of the intervention. However, these changes would be expected to influence both control and intervention groups equally within a randomised study. One of the most important changes in dietary intake of population is the decrease in total energy influenced by ageing process [40]*.* An observational study (SUN, *Seguimiento Universidad de Navarra* study) [41] carried out in Spanish population reported an average decreased energy consumption in both sexes nearly to 2.7% after 10 years of follow-up. Another study which assessed the diet quality and ND in subjects with metabolic syndrome also showed a similar energy decrease trend [42]. For this reason, we estimated the ND of the diet, which is the ratio of nutrients to the food's energy content for the amount that is commonly consumed. Our results showed that changes in energy intake are much larger than in those reported in the aforementioned observational studies, thus the dietary intervention had an effect on this issue rather than a simple fact derived from ageing process.

Finally, we are conscious about the effect of non-dietary characteristics as health status on the influence of food intake. However, at baseline both groups were comparable in all the sociodemographic and health variables analysed. For this reason, the effect observed should be attributable to the nutritional intervention performed instead of a possible modifier effect exerted by the variables mentioned previously.

Notwithstanding the above limitations, our study includes several strengths that reinforce the results obtained. Standing out the large sample size of coronary patients (*n* = 802), the use of a standardized protocol which reduces the possibility of information bias about food intake and the vast amount of sociodemographic/lifestyles variables collected in both groups. Finally, in the CORDIOPREV study, the same participants were evaluated over a period of time (7 years of follow-up). Therefore, the observed changes are less likely to be the result of differences in the sample characteristics.

## Conclusion

In conclusion, Dietary counselling after 7 years of follow-up with two healthy diets in persons at cardiovascular secondary prevention improved quality of diet, reducing total energy intake at the same time that there is a mean increasing on the intake of healthy food groups and overall ND compared to baseline. Our results reinforce the idea of the participation in trials, enhance nutrition literacy and produces better nutritional outcomes in adult patients with established CVD.

## Data Availability

Data described in the manuscript, code book, and analytic code will not be made available because of study`s embargo.

## Appendix

See Appendix Table 4.

**Table 4:** Characteristics of the participants without data at 7 years, compared to those who completed the follow-up period at the CORDIOPREV study (*n* = 802 vs. *n* = 200)

	Study sample		Lost of follow-up		p value
*n*	802		200		
	*n*	(%)	*n*	(%)	
Sex					0.514
Men	673	(83.9)	164	(82.0)	
Women	129	(16.1)	36	(18.0)	
Family history of premature CHD					0.543
No	680	(84.8)	173	(86.5)	
Yes	122	(15.2)	27	(13.5)	
Smoking habit					0.051
Never smoker	224	(27.9)	41	(20.5)	
Former smoker	504	(62.8)	133	(66.5)	
Current smoker	74	(9.2)	26	(13.0)	
Hypertension					0.073
No	263	(32.8)	51	(26.2)	
Yes	539	(67.2)	144	(73.9)	
Dyslipidaemia					0.182
LdL < 100 mg/dL	559	(69.7)	149	(74.5)	
LdL > = 100 mg/dL	243	(30.3)	51	(25.5)	
Diabetes					0.105
No	380	(47.4)	82	(41.0)	
Yes	422	(52.6)	118	(59.0)	
Marital status					0.811
Married	692	(87.4)	176	(88.0)	
Others^a^	99	(12.5)	24	(12.0)	
Missing value	1	(0.1)			
Educational level					< 0.005
Primary education	544	(70.0)	169	(84.5)	
Secondary education	164	(21.1)	25	(12.5)	
University education	69	(8.9)	6	(3.0)	

*n*	802		200		*p* value
	Mean	(SD)	Mean	(SD)	
Age (years)	59.2	(8.7)	61.2	(8.8)	0.664
BMI (kg/m^2^)	31.1	(4.5)	31.3	(4.7)	0.368
Adherence to MedDiet (points)	8.8	(1.9)	8.6	(2.0)	0.158
Adherence to low-fat diet (points)	3.9	(1.6)	3.7	(1.6)	0.470
Physical activity (METs-min/day)	190.0	(225.4)	151.6	(207.8)	0.167

Values are presented as means ± SD for continuous variables and *n* (%) for categorical variables. Pearson’s *χ*^2^ test was performed for categorical variables and *T* student test for continuous variables

*BMI*: Body Mass Index

Others^a^: Includes single, widowed and divorced

## Abbreviations

ANOVA: Analysis of variance
BMI: Body Mass Index
CORDIOPREV: CORonary Diet Intervention with Olive oil and cardiovascular PREVention study
CVD: Cardiovascular disease
FFQ: Food frequency questionnaire
IRB: Institutional review board
MEDAS: Mediterranean diet adherence score
MedDiet: Mediterranean diet
MUFA: Monounsaturated fatty acids
ND: Nutrient density
PUFA: Polyunsaturated fatty acids
SD: Standard deviation
WC: Waist circumference
