# Correction to: Long‑term effect of a dietary intervention with two‑healthy dietary approaches on food intake and nutrient density in coronary patients: results from the CORDIOPREV trial

**DOI:** 10.1007/s00394-022-03006-7

**Published:** 2022-10-03

**Authors:** Naomi Cano-Ibáñez, Gracia M. Quintana-Navarro, Juan F. Alcala-Diaz, Oriol A. Rangel-Zuñiga, Antonio Camargo, Elena M. Yubero-Serrano, Isabel Perez-Corral, Antonio P. Arenas-de Larriva, Antonio Garcia-Rios, Pablo Perez-Martinez, Javier Delgado-Lista, Jose Lopez-Miranda

**Affiliations:** 1 Department of Preventive Medicine and Public Health, University of Granada, 18071 Granada, Spain; 2 Consortium for Biomedical Research in Epidemiology and Public Health (CIBERESP), 28029 Madrid, Spain; 3 Instituto de Investigación Biosanitaria ibs.GRANADA, Complejo Hospitales Universitarios de Granada/Universidad de Granada, 18071 Granada, Spain; 4 Lipids and Atherosclerosis Unit, Internal Medicine Unit, Reina Sofia University Hospital, Av. Menendez Pidal s/n, 14004 Cordoba, Spain; 5 Maimonides Biomedical Research Institute of Cordoba (IMIBIC), 14014 Cordoba, Spain; 6 Department of Medical and Surgical Sciences, Faculty of Medicine and Nursing, University of Cordoba, 14014 Cordoba, Spain; 7 CIBER Fisiopatologia de la Obesidad y Nutricion (CIBEROBN), Instituto de Salud Carlos III, 28029 Madrid, Spain

## Correction to: European Journal of Nutrition (2022) 61:3019–3036 10.1007/s00394-022-02854-7

The original version of this article unfortunately contained errors in Table 2 in the rows 1-year and 7-year-change of the section White meat, mean (SD) (g/day).

The corrected Table 2 is given in the following page.

**Table 2:** Mean values and mean changes in dietary sources according to dietary pattern at baseline and after 1 and 7 years of follow-up in the CORDIOPREV study (*n* = 802)

	MedDiet	Low-fat diet	*p* value between group
Vegetable, mean (SD) (g/day)			
Baseline	261.8 (94.5)	254.3 (99.4)	0.276
1-year change (95% CI)	− 16.0 (− 27.1, − 5.0)	0.9 (− 11.4, 13.3)	**0.044**
7-year change (95% CI)	46.1 (35.4, 56.7)	18.1 (6.0, 30.2)	**0.001**
*p* value within group	< **0.001**	< **0.001**	
Fruits, mean (SD) (g/day)			
Baseline	346.3 (181.3)	343.2 (191.2)	0.815
1-year change (95% CI)	39.3 (17.4, 61.3)	27.7 (6.8, 48.7)	0.456
7-year change (95% CI)	121.3 (99.7, 142.8)	72.9 (50.3, 95.5)	**0.002**
*p* value within group	< **0.001**	< **0.001**	
Legumes, mean (SD) (g/day)			
Baseline	22.6 (11.8)	23.9 (17.3)	0.206
1-year change (95% CI)	3.4 (1.7, 5.1)	− 1.5 (− 3.7, 0.7)	**0.001**
7-year change (95% CI)	4.3 (2.9, 5.8)	0.2 (− 1.9, 2.2)	**0.001**
*p* value within group	< **0.001**	< **0.001**	
Dairy, mean (SD) (g/day)			
Baseline	387.0 (191.5)	351.3 (186.8)	**0.008**
1-year change (95% CI)	− 27.9 (− 47.2, − 8.6)	15.2 (− 3.6, 34.1)	**0.002**
7-year change (95% CI)	− 68.6 (− 88.8, − 48.3)	− 24.3 (− 46.1, − 2.5)	**0.004**
*p* value within group	< **0.001**	< **0.001**	
Bakery and sweets, mean (SD) (g/day)			
Baseline	26.9 (24.7)	27.4 (26.0)	0.793
1-year change (95% CI)	− 9.8 (− 12.2, − 7.4)	0.2 (− 3.0, 3.3)	< **0.001**
7-year change (95% CI)	− 15.8 (− 18.1, − 13.6)	− 13.2 (− 15.9, − 10.4)	0.134
*p* value within group	< **0.001**	< **0.001**	
Cereals, mean (SD) (g/day)			
Baseline	176.8 (80.5)	182.8 (86.1)	0.310
1-year change (95% CI)	− 23.3 (− 32.2, − 14.4)	− 35.1 (− 45.2, − 25.0)	0.085
7-year change (95% CI)	− 36.9 (− 45.7, − 28.0)	− 41.3 (− 51.7, − 31.0)	0.515
*p* value within group	< **0.001**	< **0.001**	
Whole cereals, mean (SD) (g/day)			
Baseline	40.1 (72.3)	43.9	0.481
1-year change (95% CI)	23.2 (14.5, 31.8)	19.4 (9.9, 29.0)	0.567
7-year change (95% CI)	6.9 (− 1.5, 15.4)	2.6 (− 7.3, 12.4)	0.507
*p* value within group	< **0.001**	< **0.001**	
Fish and seafood, mean (SD) (g/day)			
Baseline	106.0 (47.2)	102.9 (46.3)	0.361
1-year change (95% CI)	− 19.3 (− 24.5, − 14.1)	− 26.3 (− 31.2, − 21.4)	0.056
7-year change (95% CI)	− 7.8 (− 12.6, − 3.0)	− 22.6 (− 27.7, − 17.6)	< **0.001**
*p* value within group	< **0.001**	< **0.001**	
Red meat, mean (SD) (g/day)			
Baseline	40.7 (31.1)	46.8 (35.5)	0.102
1-year change (95% CI)	− 23.0 (− 25.9, − 20.1)	− 23.8 (− 27.3, − 20.3)	0.737
7-year change (95% CI)	− 24.0 (− 27.1, − 21.0)	− 25.9 (− 29.5, − 22.3)	0.439
*p* value within group	< **0.001**	< **0.001**	
White meat, mean (SD) (g/day)			
Baseline	69.7 (34.4)	72.3 (37.5)	0.304
1-year change (95% CI)	− 5.7 (− 9.2, − 2.2)	− 9.7 (− 13.5, − 6.0)	0.128
7-year change (95% CI)	− 9.2 (− 12.8, − 5.5)	− 17.2 (− 21.1, − 13.2)	0.004
*p* value within group	< **0.001**	< **0.001**	
Nuts, mean (SD) (g/day)			
Baseline	9.3 (10.7)	8.5 (10.5)	0.289
1-year change (95% CI)	2.1 (0.8, 3.3)	− 3.8 (− 4.8, − 2.7)	< **0.001**
7-year change (95% CI)	7.3 (5.9, 8.7)	− 3.7 (− 4.9, − 2.5)	< **0.001**
*p* value within group	< **0.001**	< **0.001**	
Olive oil, mean (SD) (g/day)			
Baseline	34.9 (12.8)	33.4 (12.3)	0.099
1-year change (95% CI)	5.2 (3.6, 6.8)	− 14.0 (− 15.5, − 12.4)	< **0.001**
7-year change (95% CI)	12.9 (11.5, 14.4)	− 11.7 (− 13.3, − 10.1)	< **0.001**
*p* value within group	< **0.001**	< **0.001**	

Values are presented as means and standard deviations (SD) at baseline point and changes (95% CI) at 1 and 7 years of follow-up. *p* value for differences between groups at 1 and 7-year follow-up using *t* test. *p* value for differences within group using ANOVA for repeated measure. Values in bald showed significant association

*CI* confidence intervals, *MedDiet* Mediterranean diet

